# Surgical resection for recurrent retroperitoneal leiomyosarcoma and liposarcoma

**DOI:** 10.1186/s12957-018-1505-4

**Published:** 2018-10-11

**Authors:** Michael J Nathenson, Constance M Barysauskas, Robert A Nathenson, William F Regine, Nader Hanna, Edward Sausville

**Affiliations:** 1 Center for Sarcoma and Bone Oncology, Dana-Farber Cancer Institute, 450 Brookline Ave, Boston, MA 02215 USA; 2 Department of Biostatistics and Computational Biology, Dana-Farber Cancer Institute, 450 Brookline Ave, Boston, MA 02215 USA; 3 University of Pennsylvania, 3440 Market Street Philadelphia, Philadelphia, PA 19146 USA; 4 University of Maryland, Greenebaum Cancer Center, South Greene Street Suite 9d10 Baltimore, Baltimore, MD 21201 USA

**Keywords:** Soft tissue sarcoma, Retroperitoneal, Leiomyosarcoma, Liposarcoma, Overall survival, Progression-free survival

## Abstract

**Background:**

Retroperitoneal soft tissue sarcomas (STS) include a number of histologies but are rare, with approximately 3000 cases in the USA per year. Retroperitoneal STS have a high incidence of local and distant recurrence. The purpose of this study was to review the University of Maryland Medical Center’s (UMMC) treatment experience of retroperitoneal STS, where the patient population served represents a diverse socioeconomic and ethnic catchment.

**Methods:**

IRB approval was obtained. We constructed a de-identified database of patients diagnosed with retroperitoneal liposarcomas (LPS) or leiomyosarcomas (LMS) treated at UMMC between 2000 and 2013. A total of 49 patients (Pts) with retroperitoneal STS met our eligibility criteria. Kaplan-Meier plots were used to graphically portray progression-free survival (PFS) and overall survival (OS). The log-rank test was used to compare time-to-event distributions.

**Results:**

The median OS for all patients (Pts) was 6.3 years, and the 2-year OS rate was 81%. The median PFS for all Pts was 1.8 years, and the 2-year PFS rate was 45%. There was no difference in OS and PFS among LMS and LPS patients; the median OS for LMS was 3.8 years vs. LPS 6.4 years (*p* = 0.33), and the median PFS for LMS was 1.2 years vs. LPS 2.5 years (*p* = 0.28). There was a significant difference between histology and race (*p* = 0.001). LPS were primarily Caucasian 86% vs. 14% black, whereas LMS were primarily black 52% vs. 33% Caucasian. OS was influenced by functional status, gender, American Joint Committee on Cancer (AJCC) stage, grade, histology, tumor size, and extent of resection. PFS was influenced by AJCC stage, grade, and extent of resection. Neither adjuvant chemotherapy (1 Pt) nor neoadjuvant/adjuvant radiation therapy (18 Pts) influenced OS or PFS. There was a non-significant difference that Pts who could undergo resection of local recurrence had improved 2-year OS, with 100% LMS and LPS compared to 2-year OS of 71% (LMS) and 78% (LPS) not undergoing resection of local recurrence.

**Conclusions:**

This study suggests a higher incidence of leiomyosarcoma in the African-American population. This study confirms the prognostic importance of grade, tumor size, AJCC stage, histology, and extent of resection in patient outcomes, at a large substantially diverse academic medical center. Future research into the biological features of liposarcoma and leiomyosarcoma Pts imparting these characteristics will be important to define.

## Background

Sarcoma is a rare cancer among adults and represents approximately 1% of all adult malignancies [1]. There are only 12,000 to 13,000 cases of adult soft tissue sarcomas (STS) per year [2]. The vast majority of STS are of the extremity or trunk, and the second largest subgroup consists of retroperitoneal sarcomas (RPS). RPS represents approximately 3000 annual cases [2] and is comprised of approximately 15 to 17% of all soft tissue sarcomas [3, 4]. The majority of retroperitoneal sarcomas are leiomyosarcomas (LMS) or liposarcomas (LPS). RPS liposarcomas consist mostly of well-differentiated and dedifferentiated liposarcomas [5, 6]. Despite improved survival rates in other cancer types, the 5-year overall survival (OS) of soft tissue sarcoma remains close to 50% [7]. For the two largest series of retroperitoneal sarcoma, the 5-year overall survival was 67% [8], the 7-year overall survival was 58%, and the 7-year disease-free survival was 38% [9].

There are several prognostic factors that can better define a patient’s survival with soft tissue sarcomas. These include size [10–13], grade [10–13], American Joint Committee on Cancer (AJCC) stage, location [13, 14], and histologic subtype [11–13] of the tumor. Prognostic factors are interconnected as the histologic subtype may influence the grade of a tumor, and the staging system is based on the size, grade, and depth of the tumor [15, 16]. Location of the tumor is also quite important, as tumors in the extremity and trunk have improved survival compared to tumors in the retroperitoneum, head, and neck, or uterus [2]. The main prognostic factors for retroperitoneal sarcomas are age, grade, tumor size, histologic subtype, tumor multifocality, and extent of resection [17]. In general, retroperitoneal sarcomas have a high rate of local recurrence and low rate of distant metastasis, although this is influenced by each histologic subtype [18–20]. For example, LPS have a high rate of local recurrence while LMS have a high rate of distant metastasis [8]. Even among retroperitoneal sarcomas of similar histology, there is variability in outcomes including recurrence and death with indolence or rapid progression with a more aggressive clinical course [21–24].

The primary management of RPS tumors remains surgical excision. The extent of resection influences patient outcomes. Patients with negative margins or R0 resection tend to have a lower risk of local recurrence. Though given the typical size and proximity to critical abdominal structures, an R0 resection is difficult to achieve in retroperitoneal sarcomas, and the majority of patients receive an R1 resection, defined as positive microscopic margins [7, 25–28]. Adjuvant or neoadjuvant radiation therapy has been shown to decrease the risk of local recurrence to approximately 10% in extremity soft tissue sarcomas, but the role of adjuvant or neoadjuvant radiation in retroperitoneal soft tissue sarcoma is still undetermined [29]. There is retrospective data that suggests a decrease in local recurrence with neoadjuvant or adjuvant radiation [30]. This benefit has not yet been confirmed in a prospective randomized trial. The role of adjuvant chemotherapy is debated in extremity soft tissue sarcomas and unknown in retroperitoneal soft tissue sarcomas [31, 32]. For patients that develop local recurrence and metastatic disease, survival is significantly worse 14 to 29% at 5 years [33]. Several nomograms have been developed to predict the outcome for patients with retroperitoneal soft tissue sarcomas using age, grade, tumor size, histology, radiation, the extent of resection, and tumor multifocality as prognostic factors [14, 17, 34, 35]. Although, only one of these nomograms has been externally validated [9].

We sought to evaluate the University of Maryland experience with retroperitoneal soft tissue sarcomas from 2000 to 2013 by examining the 2-year overall survival and progression-free survival based on patient, tumor, and treatment characteristics. Patient characteristics included sex, race, and age. Tumor characteristics included size, grade, depth, stage, location, and histologic subtype. Treatments examined included the extent and number of surgical resections, as well as the use of neoadjuvant or adjuvant radiation and chemotherapy. The influence of surgery on survival for patients with primary metastatic disease or recurrent disease was examined as well. The University of Maryland Medical Center in Baltimore services a local traditionally medically underserved catchment with a high African-American population. The patient population may be distinct from that of other major sarcoma centers with a national draw and is therefore of interest to consider.

## Methods

With approval by the University of Maryland Institutional Review Board, a de-identified database was constructed of all patients diagnosed with sarcomas treated at UMMC between 2000 and 2013. Our inclusion criteria included 28 retroperitoneal LPS and 21 retroperitoneal LMS patients (49 total).

The diagnosis of retroperitoneal LPS or LMS was confirmed by University of Maryland pathologists. Demographic, clinicopathologic, and treatment characteristics were extracted from the patient’s medical records. Stage was assigned using the AJCC 7th edition TNM soft tissue sarcoma staging system [36]. Histologic subtypes were categorized per WHO classification. Grade was determined using the FNCLCC system [15]. Performance status was determined by the Eastern Cooperative Oncology Group performance score [37].

Patient’s demographic and clinical characteristics were descriptively examined. Categorical variables were summarized in frequency tables using Fisher’s exact test, and continuous variables were summarized using median (range) and the Wilcoxon rank sum test. Kaplan-Meier plots were used to graphically portray progression-free survival (PFS) and overall survival (OS) [38]. The log-rank test was used to compare time-to-event distributions. OS was defined as the time from the date of pathologic diagnosis to death or date last known alive. PFS was defined as the time from the date of pathologic diagnosis to the date of disease progression or death. Patients without an event were right-censored at the date of last follow-up [39].

## Results

### Patient characteristics

A total of 49 LPS and LMS patients characterized the study population. LPS included both well- and dedifferentiated subtypes. Patient and tumor characteristics by histologic subtype are described in Table 1, as well as the *p* value from the log-rank test comparing the LMS and LPS populations. Median age was not significantly different between LMS (57 years, range 38–85) vs. LPS (55 years, range 39–78, *p* = 0.93). We identified a statistically significant difference between LMS and LPS patients by race, with a higher frequency of black LMS patients (52%,) compared to Caucasians (33%), and the reverse was true for LPS with Caucasian (86%) and black (14%, *p* = 0.001). Functional status was predominantly comprised of patients with an ECOG performance score of 0 to 1 and was not significantly different between LMS and LPS (*p* = 0.07). The most common presenting symptom was pain for both LMS (71%) and LPS (57%), though LPS did have a higher incidence of patients presenting with a painless enlarge mass (7%) or incidental finding on imaging (14%). The median length of presenting symptoms was similar between LMS (1.0 month, range 0 to 12 months) and LPS (1.1 months, range 0 to 12 months, *p* = 0.57).

**Table 1:** Patient characteristics

	Leiomyosarcoma (*n* = 21)	Liposarcoma (*n* = 28)	*p* value
Age at diagnosis			
Median (range)	57 (38–85)	55 (39–78)	0.93
Gender, *n* (%)			
Male	9 (43%)	18 (64%)	0.16
Female	12 (57%)	10 (36%)	
Race, *n* (%)			
Non-Hispanic white	7 (33%)	24 (86%)	0.001
Hispanic	1 (5%)	0 (0%)	
Black	11 (52%)	4 (14%)	
Asian	1 (5%)	0 (0%)	
Unknown	1 (5%)	0 (0%)	
ECOG functional status, *n* (%)			
0	10 (48%)	9 (32%)	0.07
1	6 (29%)	17 (61%)	
2	2 (9%)	0 (0%)	
Unknown	3 (14%)	2 (7%)	
Initial presenting symptom, *n* (%)			
Bone/abdomen/back/flank/groin pain	15 (71%)	16 (57%)	0.57
Enlarging painless mass	0 (0%)	2 (7%)	
Incidental finding on imaging/examination	1 (5%)	4 (14%)	
Other	2 (10%)	4 (14%)	
Unknown	3 (14%)	2 (7%)	
Length of presenting symptoms (*n* = 28)			
Median (range)	1.0 (0–12)	1.1 (0–12)	0.57
Length of presenting symptoms			
≤ 2 months	8 (38%)	12 (43%)	0.93
> 2 months	4 (19%)	4 (14%)	
Unknown	9 (43%)	12 (43%)	
Histologic grade, *n* (%)			
1	2 (9%)	14 (50%)	0.002
2	6 (29%)	1 (4%)	
3	10 (48%)	12 (43%)	
Unknown	3 (14%)	1 (4%)	
Tumor size (cm) [*n* = 39]			
Median (range)	10.4 (3–29)	27.3 (6–54)	0.0001
Tumor size (cm)			
< 15 cm	13 (62%)	3 (11%)	< 0.0001
≥ 15 cm	2 (9%)	21 (75%)	
Unknown	6 (29%)	4 (14%)	
Stage, *n* (%)			
1	1 (5%)	12 (43%)	0.002
2	6 (29%)	1 (4%)	
3	7 (33%)	11 (39%)	
4	4 (19%)	1 (4%)	
Unknown	3 (14%)	3 (11%)	
Resection status, *n* (%)			
No residual disease (R0)	11 (52%)	12 (43%)	0.49
Microscopic disease (R1)	4 (19%)	11 (39%)	
Gross residual disease (R2)	1 (5%)	2 (7%)	
No surgery (biopsy only)	3 (14%)	2 (7%)	
Unknown	2 (10%)	1 (4%)	
Neoadjuvant or adjuvant radiation			
No	9 (43%)	17 (60%)	0.20
Yes	8 (38%)	10 (36%)	
Unknown	4 (19%)	1 (4%)	
Recurrent disease, *n* (%)			
No	5 (24%)	7 (25%)	0.56
Yes	14 (67%)	15 (54%)	
Unknown	2 (9%)	6 (21%)	
Local recurrence, *n* (%)			
No	12 (57%)	7 (25%)	0.03
Yes	4 (19%)	14 (50%)	
Not applicable	3 (14%)	1 (4%)	
Unknown	2 (10%)	6 (21%)	
Resection of local recurrence, *n* (%)			
No	14 (67%)	9 (32%)	0.02
Yes	2 (10%)	12 (43%)	
Unknown	5 (24%)	7 (25%)	
Cause of death, *n* (%)			
Metastatic or recurrent sarcoma	7 (33%)	8 (29%)	0.79
Heart failure	1 (5%)	0 (0%)	
Still alive	9 (43%)	15 (53%)	
Unknown	4 (19%)	5 (18%)	
Alive, *n* (%)			
Dead	12 (57%)	12 (44%)	0.56
Alive	9 (43%)	15 (56%)	

Tumor grade, size, and stage significantly differed between LMS and LPS. The median tumor size for LMS was 10.4 cm (range 3 to 29 cm) compared to LPS 27.3 cm (range 6 to 54 cm), *p* = 0.0001. Additionally, only 9% of LMS patients were considered grade 1, while 50% of LPS patients had grade 1 tumors (well-differentiated without a dedifferentiated component), (*p* = 0.002). There was a higher rate of grade 2 or intermediate grade tumors for LMS 29% vs. only 4% for LPS. The percentage of tumors that were high grade was equivalent between LMS (48%) and LPS (43%). The sarcoma staging system is based on the size and grade. There was a lower percentage of stage I tumors in LMS (5%) compared to stage I tumors in LPS, (43%, *p* = 0.002).

Recurrent disease occurred in 67% of LMS patients and 54% of LPS patients (*p* = 0.56). Local recurrence was lower in patients with LMS (19%) than in LPS (50%, *p* = 0.03). The majority of LMS patients developed distant metastasis; this included lung (9 patients), liver (7 patients) bone (2 patients), and soft tissue (1 patient). Of 15 LPS patients with recurrence, only 3 developed distant metastases, liver (1 patient), and soft tissue (2 patients).

### Patient treatments

The majority of LMS and LPS patients underwent surgical resection, though three LMS patients and two LPS patients underwent biopsy alone. For LMS and LPS patients that underwent surgery, there was a high rate of R1 and R2 resections. For LMS, R1 is 19% and R2 is 5%, compared to LPS, R1 resection 39% and R2 resection 7%. There was only one patient that underwent adjuvant chemotherapy with vincristine, doxorubicin, and ifosfamide, an LPS patient. The dosing and rationale for this regimen were not documented. However, there were 18 patients that received neoadjuvant or adjuvant radiation therapy, eight LMS patients and ten LPS patients. Twenty-six patients did not receive radiation, and the radiation status of five patients was unknown. The mean radiation dose was 54.2 Gy, with the majority of patients receiving external beam/intensity-modulated radiation therapy (IMRT). Neoadjuvant or adjuvant radiation did not significantly affect OS (*p* = 0.65) or PFS (*p* = 0.69). Fewer patients with LMS underwent resection for recurrent disease 10% vs. 43% for LPS, *p* = 0.02.

### Overall survival

**Fig. 1:**
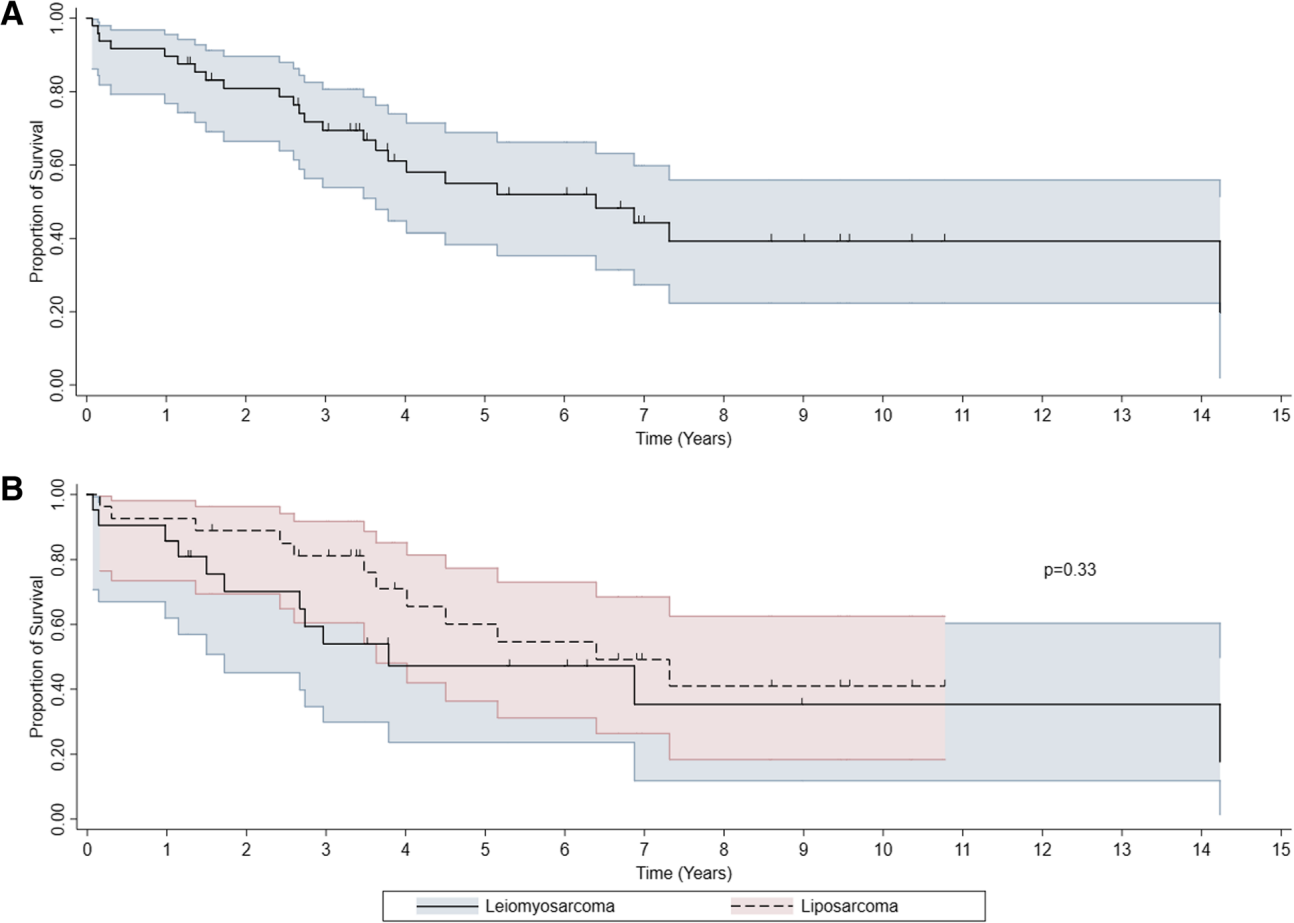
**a** Kaplan-Meier overall survival. **b**. Kaplan-Meier overall survival by LMS vs. LPS. The shaded lines indicate the confidence interval of overall survival

At the time of the last follow-up, 24 patients were alive, and median follow-up for patients still alive was 6.9 years (95% CI 3.8–9.4). As of July 1, 2014, among our cohort of 49 patients, there were a total of 25 deaths including 12 deaths among LMS patients (57%) and 12 deaths among LPS patients (44%). The median overall survival from date of pathologic diagnosis was 6.3 years (95% CI 3.6–∞, Fig. 1a) and the 2-year OS rate 81% (95% CI 66–90%). The median overall survival from date of pathologic diagnosis among LMS patients was 3.8 years (95% CI 1.7–14.2) and 6.4 years (95% CI 3.6–∞) among LPS patients (*p* = 0.33, Fig. 1b). The 2-year OS rates were 70% (95% CI 45–85%) and 89% (95% CI 69–96%) among LMS and LPS patients, respectively. Table 2 demonstrates the 2-year OS rate and the log-rank test summary comparing OS by patient, tumor, and treatment characteristics to identify possible prognostic factors.

**Table 2:** Two-year overall survival log-rank summary

	2-year OS: leiomyosarcoma (%)	2-year OS: liposarcoma(%)	*p* value
Race			0.69
Non-Hispanic white	71	88	
Hispanic	100	–	
Black	61	100	
Asian	100	–	
Unknown	100	–	
Gender			0.05
Female	82	100	
Male	56	82	
Functional status			0.0004
0	79	89	
1	63	88	
2	88	–	
Unknown	100	100	
Stage			< 0.0001
1	100	92	
2	100	100	
3	57	91	
4	25	–	
Unknown	100	100	
Grade			0.05
1	100	92	
2	100	100	
3	60	83	
Unknown	33	100	
Age, years			0.22
≤ 55	80	100	
> 55	58	75	
Length of presenting symptoms, months			0.69
≤ 2	63	83	
> 2	75	100	
Unknown	74	91	
Tumor size (cm)			< 0.0001
< 15	75	67	
≥ 15	–	85	
Unknown	83	67	
Resection status			< 0.0001
R0	100	100	
R1	50	91	
R2	–	50	
Biopsy/none	–	50	
Unknown	100	100	
Radiation therapy			0.65
No	53	88	
Yes	88	90	
Unknown	67	100	
Resection of local recurrence, *n* (%)			0.88
No	71	78	
Yes	100	100	
Unknown	60	86	

### Progression-free survival

**Fig. 2:**
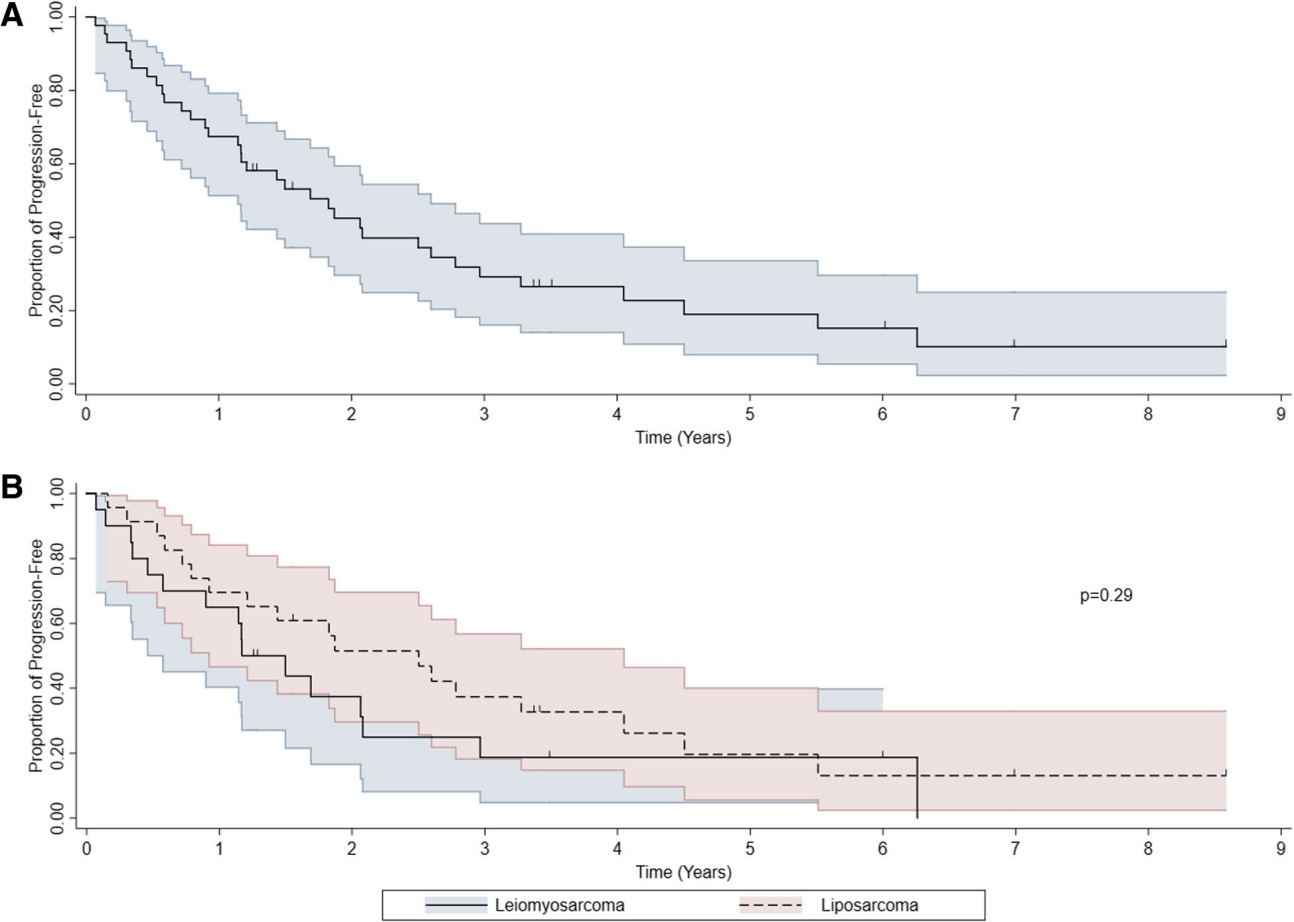
**a**. Kaplan-Meier progression-free survival. **b** Kaplan-Meier progression-free survival by LMS vs. LPS. The shaded lines indicate the confidence interval of progression-free survival

Among our cohort of 43 patients (6 patients were excluded from the analysis due to unknown progression information), there were a total of 34 events. A total of 9 patients progressed but did not die during follow-up, and 24 patients progressed and died during follow-up. The median progression-free survival was 1.8 years (95% CI 1.1–2.6, Fig. 2a). The 2-year PFS rate was 45% (95% CI 30–59%). There was a trend towards improved progression-free survival with LPS patients compared to LMS patients. The median progression survival among leiomyosarcoma patients was 1.2 years (95% CI 0.5–2.1) and 2.5 years (95% CI 0.9–4.0) among liposarcoma patients (*p* = 0.29, Fig. 2b). The 2-year PFS rates were 38% (95% CI 17–59%) and 52% (95% CI 30–70%) among leiomyosarcoma and liposarcoma patients, respectively. Table 3 demonstrates the 2-year PFS rate and the log-rank test summary comparing PFS by patient, tumor, and treatment characteristics to identify possible prognostic factors.

**Table 3:** Two-year progression-free survival log-rank summary

	2-year PFS: leiomyosarcoma (%)	2-year PFS: liposarcoma (%)	*p* value
Race			0.92
Non-Hispanic white	29	49	
Hispanic	100	–	
Black	27	67	
Asian	–	–	
Unknown	100	–	
Gender			0.49
Female	42	57	
Male	33	49	
Functional status			0.27
0	42	63	
1	44	42	
2	–	–	
Unknown	33	100	
Stage			0.002
1	–	60	
2	75	–	
3	14	42	
4	25	–	
Unknown	33	100	
Grade			0.04
1	100	64	
2	75	–	
3	20	38	
Unknown	–	100	
Age, years			0.29
≤ 55	30	27	
> 55	45	75	
Length of presenting symptoms, months			0.09
≤ 2	38	80	
> 2	–	33	
Unknown	50	30	
Tumor size (cm)			0.15
< 15	47	50	
≥ 15	–	55	
Unknown	33	33	
Resection status			< 0.0001
R0	61	62	
R1	–	44	
R2	–	–	
Biopsy/none	–	50	
Unknown	100	–	
Radiation therapy			0.69
No	33	49	
Yes	38	56	
Unknown	50	–	
Resection of local recurrence, *n* (%)			0.31
No	32	67	
Yes	50	42	
Unknown	50	50	

### Prognostic factors

**Fig. 3:**
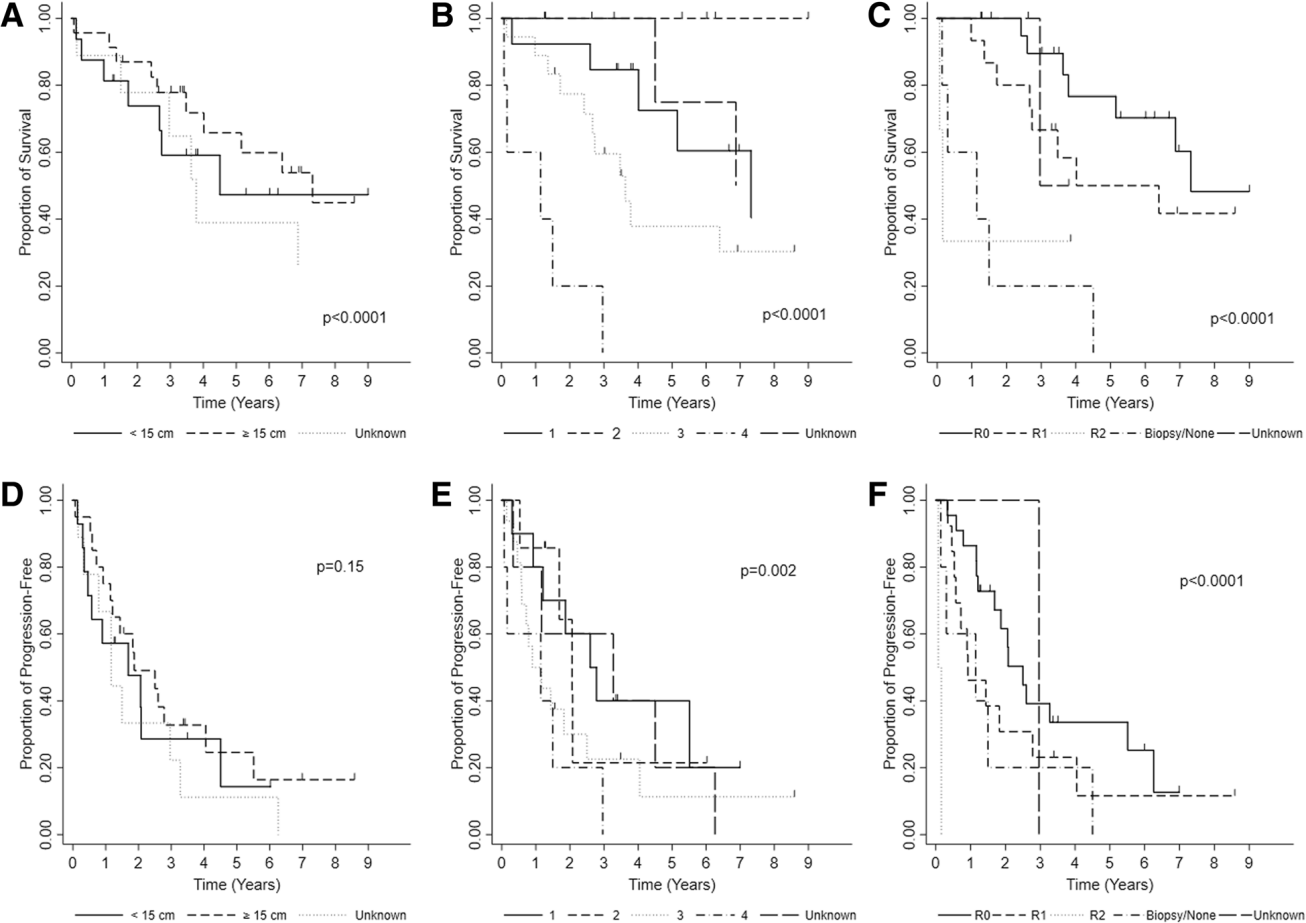
Prognostic factors for overall survival **a** initial tumor size **b** stage **c**) extent of resection. Prognostic factors for progression-free survival **d** initial tumor size **e** stage **f** extent of resection

Prognostic factors that influenced overall survival were gender (*p* = 0.05), performance status (*p* = 0.0004), grade (*p* = 0.05), tumor size (< 0.0001) (Fig. 3a), stage (< 0.0001) (Fig. 3b), and extent of resection (*p* < 0.0001) (Fig. 3c). The prognostic factors that influenced progression-free survival were stage (*p* = 0.002) (Fig. 3e), grade (*p* = 0.04), and extent of resection (*p* < 0.0001) (Fig. 3f), which correspond to the overall survival prognostic factors. Two patients with LMS underwent resection of local recurrence, and 12 patients with LPS underwent resection of local recurrence. There was a trend that patients who underwent resection of local recurrence had improved 2-year overall survival LMS (100%) and LPS (100%) compared to patients that did not undergo resection of local recurrence LMS (71%) and LPS (78%), though this difference was not statistically significant (*p* = 0.88).

### Health disparities

There was a higher percentage of LMS patients that were black compared to Caucasian, yet the 2-year overall survival was worse. black patients with LMS had a 61% 2-year overall survival compared to Caucasian 71% 2-year overall survival. The reverse was true for LPS, with black LPS patients attaining a 100% 2-year overall survival compared to Caucasians with 88% 2-year overall survival. There were no other socioeconomic factors available for retrospective analysis.

## Discussion

Sarcomas are a group of rare diseases. The most common retroperitoneal sarcomas are LMS and LPS [2]. However, the natural history of these diseases differs greatly. LMS vary from slowly growing, indolent to very aggressive neoplasms [40]. By definition, these tumors are usually classified and intermediate to high grade [40]. LMS tend to have a higher rate of metastatic disease [40]. There are four different liposarcoma subtypes, the most common being well-differentiated LPS and dedifferentiated LPS, and the rarer subtypes include myxoid LPS and pleomorphic LPS, which occur less often in the retroperitoneum [41]. It is very common for retroperitoneal LPS to have both well-differentiated and dedifferentiated components [40]. Well-differentiated LPS are low-grade tumors, slowly growing, and minimally symptomatic [41]. These tumors can grow to a very large size before diagnosis, and R0 resections are uncommon. The rate of local recurrence alone for retroperitoneal LPS is greater than those with distant metastasis. Dedifferentiated LPS may be extremely aggressive [41].

These variances in retroperitoneal sarcoma natural history for both LMS and LPS are exemplified in this patient population. The LPS in this population have a higher rate of low-grade tumors, larger tumor size, and therefore higher percentage of stage I tumors. Not unexpectedly, there was a high rate of R1 and R2 resections for LPS. This is compared to the LMS population, with mostly intermediate to high-grade tumors and smaller size. Furthermore, the local recurrence rate was higher in LPS patients, and the distant recurrence rate was higher in LMS. This study showed the 2-year overall survival of 81% and 2-year progression-free survival of 45%. There was a trend towards improved survival with LPS compared to LMS, as has been previously reported [8, 9]. This trend was not statistically significant in this study but may have been influenced by the small sample size in this population.

However, one key difference of interest in the UMMC sarcoma population compared to other sarcoma centers is race, with 30% of the population black, a higher percentage of the population than reported in similar retroperitoneal sarcoma studies [8, 9]. Racial disparities in treatment and outcomes for soft tissue sarcomas have been previously noted, with black patients more likely to die of their disease [42, 43]. Our study suggests a difference in the incidence of retroperitoneal LMS and LPS by race, as most of the black patients in this population had LMS, and not LPS. This difference has not been previously noted and is worth examining in a larger patient population. Furthermore, the overall survival for black patients with leiomyosarcoma was decreased compared to Caucasian patients. This difference was not statistically significant; however, the small sample size of this population does limit statistical analysis. Larger studies of outcomes in soft tissue sarcomas have noted a correlation between race and socioeconomic status [42, 43]. In the UMMC population, socioeconomic status was difficult to quantify in a retrospective study, as family status, occupation, and income were not universally available in a retrospective record review. However, UMMC is located in Baltimore, MD, addressing the needs of a large underserved and uninsured black population. Race could be a marker for socioeconomic status. The impact of race and socioeconomic status on treatment outcomes should be examined in a larger cohort of retroperitoneal sarcoma patients.

The standard of care management of retroperitoneal sarcoma is surgical resection alone, though there is a high rate of R1 or R2 resections [23, 25, 26]. The role of chemotherapy is uncertain. Adjuvant or neoadjuvant chemotherapy is used by some high-volume sarcoma centers; however, there is no randomized controlled evidence to support the use of chemotherapy for retroperitoneal sarcomas in the curative setting [44]. Only one patient in this study received adjuvant chemotherapy. A role of adjuvant or neoadjuvant chemotherapy for retroperitoneal sarcomas cannot be recommended at this time. Adjuvant or more commonly neoadjuvant radiation is used in the treatment of retroperitoneal sarcomas to decrease the risk of local recurrence. Neoadjuvant radiation is preferred as the dose of radiation is lower, the radiation field is smaller, and abdominal structures, including bowel, can be sparred toxicity. The benefit of radiation may differ among retroperitoneal sarcoma histologies such as LMS vs. well-differentiated or dedifferentiated LPS. This study shows no benefit to adjuvant or neoadjuvant radiation, similar to previous retrospective reports [45]. However, there is a prospective randomized trial (NCT01344018) of surgery vs. neoadjuvant radiation and surgery for retroperitoneal sarcomas, which hopefully will provide more definitive data for the efficacy or lack thereof of neoadjuvant radiation for retroperitoneal sarcomas. At the moment, neoadjuvant radiation can be considered in the treatment of retroperitoneal sarcoma on a case-by-case basis. The best management for recurrent retroperitoneal sarcomas is uncertain.

Three retroperitoneal sarcoma-specific nomograms have been published. These nomograms are designed to help predict prognosis for retroperitoneal sarcoma patients. The Anaya et al. nomogram uses age, size, multifocality, primary vs. recurrence, and completeness of resection as prognostic factors [34]. The Tan et al. nomogram uses size radiation, histology, and extent of resection as prognostic factors [35]. The Gronchi et al. nomogram was externally validated and uses grade, size, age, histology, extent of resection, and multifocality as prognostic factors [9, 14]. This study provides further evidence that supports the importance of histology, extent of resection, grade, size, and therefore stage in the prognosis of retroperitoneal leiomyosarcomas and liposarcomas. Our study also suggests that race may be an underappreciated prognostic factors and although limited by its small sample size points to the need for a better understanding of racial and socioeconomic disparities in retroperitoneal sarcomas that might be possible in a larger cohort.

Previous studies have suggested a benefit of resection of recurrent disease [46]. Our study shows a trend towards improved survival for patients that underwent resection for recurrent disease, albeit not statistically significant. It is also true that more patients with LPS underwent resection for recurrent disease than LMS patients, likely influenced by the higher rate of local recurrence in LPS compared to LMS. In concordance with previous reports [46], our results suggest the strategic use of multiple successive resections when clinically feasible and emphasize that Pts able to undergo palliative surgery may attain superior survival.

## Conclusions

Retroperitoneal sarcoma is a rare subset of cancer, most commonly including liposarcomas and leiomyosarcoma. Our study suggests a higher incidence of leiomyosarcoma in the African-American population. The standard of care treatment is surgical resection, though the role of neoadjuvant radiation is being examined. The most important prognostic factors for retroperitoneal LPS and LMS are the extent of resection, grade, and size, and race may be an important prognostic factor as well. This study was likely limited by small sample size, but our experience underscores the need for recurrent retroperitoneal sarcoma patients to be evaluated by a center experienced in undertaking extensive surgery for recurrent disease if clinically feasible.

## Abbreviations

AJCC: American Joint Committee on Cancer
CI: Confidence interval
FNCLCC: Federation Nationale des Centers de Lutte Contre le Cancer
IMRT: Intensity-modulated radiation therapy
IRB: Institution review board
LMS: Leiomyosarcoma
LPS: Liposarcoma
OS: Overall survival
PFS: Progression-free survival
Pts: Patients
RPS: Retroperitoneal sarcomas
STS: Soft tissue sarcoma
TNM: Tumor node metastasis
UMMC: University of Maryland Medical Center
WHO: World Health Organization
